# Accuracy of Patient‐Reported Exposure to New Psychoactive Substances and Other Illicit Drugs in Australian Emergency Departments: Findings From the Emerging Drugs Network of Australia

**DOI:** 10.1111/dar.70193

**Published:** 2026-06-15

**Authors:** Marjan J. Nijmeijer, Jennifer L. Smith, Courtney Weber, Bas W. J. Bens, Daniel M. Fatovich, Shaun L. Greene, Jennifer L. Schumann, Paul Dessauer, Rebekka Syrjanen, Katherine Z. Isoardi, Keith Harris, Sam Alfred, Viet Tran, Ewoud ter Avest

**Affiliations:** ^1^ Department of Acute Care University Medical Centre Groningen, University of Groningen Groningen the Netherlands; ^2^ Medical School, The University of Western Australia Perth Australia; ^3^ Centre for Clinical Research in Emergency Medicine Harry Perkins Institute of Medical Research Perth Australia; ^4^ East Metropolitan Health Service Perth Australia; ^5^ School of Population and Global Health The University of Western Australia Perth Australia; ^6^ Emergency Medicine Royal Perth Hospital, University of Western Australia Perth Australia; ^7^ Department of Emergency Medicine Austin Hospital Melbourne Australia; ^8^ Victorian Poisons Information Centre, Austin Health Melbourne Australia; ^9^ Department of Critical Care University of Melbourne Melbourne Australia; ^10^ Victorian Institute of Forensic Medicine Melbourne Australia; ^11^ Department of Forensic Medicine Monash University Melbourne Australia; ^12^ Monash Addiction Research Centre, Monash University Melbourne Australia; ^13^ Peer Based Harm Reduction WA Perth Australia; ^14^ National Drug and Alcohol Research Centre UNSW Sydney Sydney Australia; ^15^ Clinical Toxicology Unit Princess Alexandra Hospital Brisbane Australia; ^16^ Faculty of Medicine University of Queensland Brisbane Australia; ^17^ Royal Adelaide Hospital Adelaide Australia; ^18^ Faculty of Medicine University of Adelaide Adelaide Australia; ^19^ Royal Hobart Hospital, Tasmanian Health Service Hobart Australia; ^20^ Tasmanian School of Medicine, University of Tasmania Hobart Australia; ^21^ Menzies Institute for Medical Research, University of Tasmania Hobart Australia; ^22^ London's Air Ambulance London UK

**Keywords:** emergency service, harm reduction, hospital, illicit drugs, new psychoactive substances, toxicology

## Abstract

**Introduction:**

New psychoactive substances (NPS) present a unique challenge in clinical, public health and drug‐policy contexts. Continued diversity, unknown potency and often unintentional exposure can limit the accuracy of self‐reported data. This study examined the accuracy of patient‐reported NPS and illicit drug exposure in Australian emergency departments (ED).

**Methods:**

Patient‐reported drug exposure, clinical and toxicology data were extracted from the Emerging Drugs Network of Australia Clinical Registry between 1 July 2021 and 30 June 2024 for patients presenting with severe and/or unusual illicit drug toxicity in the ED. Blood samples were analysed using mass spectrometry. Agreement between reported and confirmed exposure was assessed using Cohen's kappa, sensitivity, specificity and likelihood ratios. Logistic regression analysis identified factors associated with discrepancies between reported and confirmed exposures.

**Results:**

There were 2044 presentations: 64.6% male, median age 33 years (Q1–Q3, 26–41). Complete agreement between patient‐reported and confirmed drug exposures was 14.3% (*n* = 293). Agreement between reported and confirmed NPS exposure was poor (*κ* = 0.38). 1522 (74.5%) had more drugs detected than reported. Older age (OR 1.03 [CI 1.02, 1.04]) was associated with higher odds of discrepancy. Attendance from a licensed venue (OR 0.44 [CI 0.28, 0.70]) and Glasgow Coma Scale of 13–15 (OR 0.44 [CI 0.33, 0.57]) were associated with lower odds.

**Discussion and Conclusions:**

Poor agreement between patient‐reported and analytically confirmed drug exposure highlights the need for continued partnerships between EDs, clinical toxicologists, and forensic laboratories to identify substances involved in acute intoxications and support public health and harm reduction responses.

## Key Points

- The potency, pharmacological effects and risk profiles of many new psychoactive substances (NPS) are largely unknown, and exposure is often unintentional.
- Complete agreement between patient‐reported and analytically confirmed drug exposures was 14.3%; 74.5% had more drugs detected than reported.
- Patient‐reporting of NPS is unreliable, with poor agreement to analytically confirmed NPS exposure (*κ* = 0.38). For traditional illicit drugs, agreement ranged from poor (*κ* = 0.25, methamphetamine) to good (*κ* = 0.73, gamma‐hydroxybutyrate).
- Toxicosurveillance data gathered from emergency department presentations can contribute to early warning systems, helping to promptly identify emerging threats and to inform timely public health and harm reduction responses.

## 1 Introduction

New psychoactive substances (NPS) are increasingly implicated in non‐fatal and fatal poisonings, and present significant challenges for clinical, public health and global regulatory responses [1, 2]. These substances are often active derivatives or analogues of ‘traditional’ illicit drugs (e.g., cocaine, heroin, methylamphetamine, 3,4‐methylenedioxymethylamphetamine [MDMA]) or of approved pharmaceutical drugs such as benzodiazepines [2, 3]. The increasing diversity of new synthetic drugs and contemporary supply chains driven by darknet markets, social media and encrypted applications [4, 5, 6] make distribution easier, and hamper efforts to control supply [1]. The rising prominence of counterfeit pharmaceuticals [7] (including products containing unpredictable mixtures of novel benzodiazepines [8] and/or novel opioids [9]) and the economic appeal to organised crime syndicates of cheaper, more easily trafficked, higher‐potency substitutes are of particular concern [10, 11].

In the emergency department (ED), management of acute intoxications is guided by presenting clinical features and, when available, drug exposure information reported by the patient and/or other sources (e.g., ambulance paramedics, bystanders, attending police). Reliable and practical tests for NPS are lacking within most hospital settings. Rapid analytical tests such as urine immunoassays are common practice, but are limited in both scope and sensitivity to accurately detect most NPS compounds [12]. Although mass spectrometry offers greater accuracy, it is expensive, time‐consuming and often restricted to offsite forensic laboratories [13].

An alternative to analytical testing is to rely on patient‐reported drug exposure. However, previous research on the accuracy of patient‐reported exposure to illicit drugs often used immunoassays as the gold standard and has yielded conflicting results [14]. Studies specifically examining the reliability of patient‐reported illicit drug and/or NPS exposure are sparse and limited in drug scope, detection methods and sample size [15, 16, 17]. This is also complicated by the unintentional nature of most NPS exposures, which typically occur via substitution or adulteration of other illicit drugs or via exposure to counterfeit pharmaceuticals [4, 18, 19].

As inaccurate self‐reporting potentially influences clinical management, this study sought to evaluate the accuracy of patient‐reported exposure to NPS and/or other illicit drugs in ED presentations with accompanying comprehensive analytical results recorded in the Emerging Drugs Network of Australia (EDNA) Clinical Registry between 1 July 2021 and 30 June 2024.

## 2 Methods

### 2.1 EDNA Study Design

EDNA is an observational, prospective toxicosurveillance system of illicit drug‐related presentations to sentinel EDs across five Australian states (Queensland, South Australia, Tasmania, Victoria and Western Australia). Purposive sampling is based on the following inclusion criteria: patients aged 16 years and over (18 years and over in South Australia), presenting with severe and/or unusual clinical features of reported or suspected recreational illicit drug toxicity, and/or patients presenting as part of a suspected cluster of illicit drug intoxications, and with a requirement for intravenous cannulation as part of standard care [20, 21]. Blood samples are collected and transferred to relevant state forensic laboratories for comprehensive analysis using mass spectrometry techniques to identify a broad range of NPS and illicit and pharmaceutical drugs. Detection capability is determined by each laboratory's in‐house testing procedures and access to certified reference materials. External databases (e.g., HighResNPS.com) are utilised for tentative compound identification if certified reference material is unavailable. Detection thresholds are adopted by each laboratory to confirm drug exposure. Detailed inclusion criteria and protocols for blood sampling and analytical testing have been previously described [21].

Ethics approval, including waiver of consent, for the establishment of a de‐identified national ED toxicosurveillance registry was granted by the South Metropolitan Health Service Human Research Ethics Committee (RGS0000003673) in December 2019. EDNA is registered with the Australian and New Zealand Clinical Trials Registry (ACTRN12621001234808) and the Clinical Registry is hosted on a secure online data management system (REDCap) by Curtin University [22].

### 2.2 Participants

**FIGURE 1:**
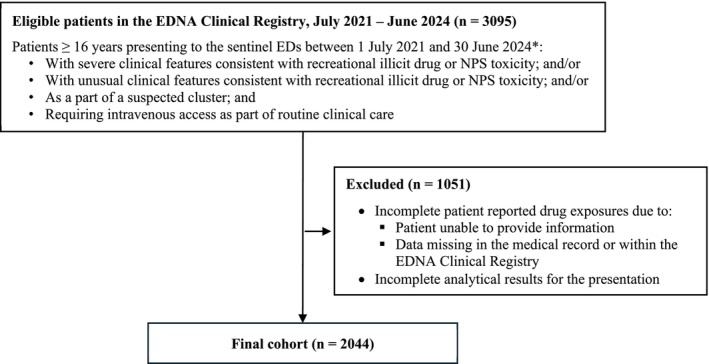
Flow chart of the study population. ED, emergency department; EDNA, emerging drugs network of Australia; NPS, new psychoactive substance. * ≥ 18 years old in South Australia.

ED presentations included in the EDNA Clinical Registry were extracted for analysis if: (i) the triage date was between 1 July 2021 and 30 June 2024; and (ii) both patient‐reported drug exposure and laboratory confirmed toxicology results were complete at the time of data extraction (26 September 2024) (Figure 1). The Clinical Registry contains two separate instruments for collecting reported drug exposure data: (i) patient‐reported; and (ii) other sources of reported drug exposure(s), including family or friends, ambulance paramedics, police, product labels, urine drug screens, other or unknown. As the aim of this study was to explore the accuracy of patient‐reported drug exposure, our analysis focuses only on documented patient self‐report information. Additional exclusion criteria for patient‐reported data are presented in Figure 1. Patients who actively denied drug exposure were categorised as ‘did not report drug use’ and were retained in the analysis when corresponding toxicology results were available.

### 2.3 Measures

The following data were extracted from the Clinical Registry for all eligible presentations: basic demographics (age and sex), patient‐reported drug exposure(s) (documented in the ambulance record and/or the medical record related to the ED presentation), ED presentation characteristics (arrival mode, Australasian Triage Scale [ATS] [23], setting of drug exposure or last known location prior to ED arrival), initial Glasgow Coma Score (GCS), clinical outcome data (ED disposition and length of stay), and laboratory confirmed toxicology results.

#### 2.3.1 Categorisation of Self‐Report and Analytical Data

Patient‐reported and analytically confirmed drug exposures per presentation were stratified into the following categories: (i) NPS; (ii) traditional illicit; and (iii) pharmaceutical. NPS were organised into effect groups consistent with the United Nations Office on Drugs and Crime classification system [2]: novel benzodiazepines; other sedatives/hypnotics; novel opioids; novel stimulants; novel dissociatives; synthetic cannabinoid receptor agonists; and unassigned novel drugs. Traditional illicit drugs comprised cocaine, gamma‐hydroxybutyrate (GHB), heroin, MDMA, methamphetamine, ketamine and hallucinogens. Heroin was defined as detection of 6‐monoacetylmorphine. Although ketamine is a legally produced and restricted substance in Australia, it was classified as a traditional illicit drug in the current analysis if there was no documented evidence of medical administration pre‐hospital and/or in the ED. Cannabis was excluded from the current analysis due to interstate variability in laboratory testing protocols (e.g., two participating states do not routinely screen for tetrahydrocannabinol).

Pharmaceutical drugs included pharmaceutical benzodiazepines, pharmaceutical opioids, pharmaceutical stimulants, pregabalin, and quetiapine (Table S1). Benzodiazepine exposure reported by a patient was considered a novel benzodiazepine if there was reference to “street” or pharmaceutical brands not sold in Australia (e.g., “Xanax”) [8]. Free‐text drug exposure information entered into the Clinical Registry as patient‐reported is presented in Table S2.

Patient‐reported and analytical results were recorded as ‘none’ and ‘no drug detected’ respectively if none of the abovementioned drug categories were reported. To avoid double‐counting of primary compounds and metabolites in the same blood sample, metabolites were either: (i) excluded when the parent drug was also present; (ii) coded as the parent drug (e.g., 8‐aminoclonazolam was recorded as clonazolam in the absence of clonazolam identification); or (iii) retained as the metabolite (e.g., MDA was retained when MDMA was not detected). We were unable to determine if detected amphetamine represented a metabolite of methamphetamine, illegally produced amphetamine/speed, or use of pharmaceutical stimulants. Therefore, we did not include data on reported amphetamine/speed in our accuracy analysis. Pharmaceutical drugs administered pre‐hospital (recorded in ambulance records) and/or in the ED for patient management were excluded from analysis.

### 2.4 Outcomes

The primary outcome was to assess the agreement between patient‐reported and analytically confirmed drug exposures. ‘Complete’ agreement was defined as presentations where *all* patient‐reported drug exposures matched *all* drugs confirmed in analytical results. ‘Drug‐type’ agreement occurred when a patient‐reported drug type matched a detected drug type. Secondary outcomes included identification of factors associated with higher odds of discrepancy between reported and analytically confirmed drug exposure. Discrepancy was defined as instances when a patient did not report the drug detected.

### 2.5 Statistical Analysis

Categorical variables were presented as frequencies and percentages; continuous variables as medians with first and third quartiles (Q1–Q3) and range. The accuracy of patient‐reported drug exposures was measured using Cohen's Kappa (*κ*) (excellent (> 0.75), fair to good (0.4–0.75), or poor (< 0.4)) [24]. Sensitivity, specificity, positive likelihood ratio (LR+), and negative likelihood ratio (LR−) with 95% confidence intervals (CI) were calculated to quantify the accuracy of patient‐reported drug exposure. Accuracy was only measured when there were more than five reported drug exposures and more than five detected drug exposures in the same effect group.

To test for differences in baseline characteristics between ED presentations with and without confirmed NPS exposure, logistic regression was used for sex, age, arrival mode, setting, ATS and initial GCS. Multinomial logistic regression was used for ED disposition and truncated negative binomial regression was used for ED length of stay and total number of drugs detected. This accounted for overdispersion and truncation at zero. Regression techniques used robust variance due to the high likelihood of repeat presentations by patients over time, and therefore the potential violation of independence assumptions. A *p*‐value of < 0.05 was considered statistically significant.

Univariate regression analysis was used to identify presentation characteristics associated with higher odds of discrepancy between reported and confirmed drug exposure. All univariate analyses with a *p*‐value < 0.2 were included in the final multivariate regression model. Given the exploratory nature of this research, no corrections for multiple testing were applied. Data processing and statistical analysis were conducted using IBM SPSS Statistics (Version 29.0.2.0.) and SAS software (Version 9.4.).

## 3 Results

### 3.1 Study Population

Between July 2021 and June 2024, 2044 ED presentations were eligible for inclusion in the current analysis, representing 66.0% of the 3095 ED presentations included in the Clinical Registry during the study period (Figure 1). The median age was 33 years (range 16–76 years) and 64.6% were male (Table 1). The majority (81.7%, 1669/2044) arrived at the ED by ambulance and over a third (37.3%, 752/2044) had a GCS of 3–8 and were triaged as high acuity presentations (ATS 1, 38.6%, 786/2044). Most were discharged home after their visit or were hospitalised in a short‐stay or observation ward (Table 1). Presentations with NPS detected were more often male (*p <* 0.001), younger (*p <* 0.001) and more likely to present from a primary residence than a public environment (*p =* 0.004) compared to presentations with no NPS detected (Table 1).

**TABLE 1:** Characteristics of the study population stratified by presence of NPS.

	Total, *n* = 2044	One or more NPS detected, *n* = 227 (88.9%)	No NPS detected, *n* = 1817 (11.1%)	*p*
Demographics				
Male sex, *n* (%)	1320 (64.6)	167 (73.6)	1153 (63.5)	< 0.001*
Missing = 1				
Age in years, median (Q1–Q3)	33 (26–41)	26 (22–33)	34 (27–42)	< 0.001*
Age in years, range	16–76	16–71	16–76	
Missing = 8				
ED presentation characteristics				
Setting, *n* (%)				0.008*
Primary residence	768 (37.7)	111 (48.9)	657 (36.2)	—
Public environment	745 (36.5)	71 (31.3)	674 (37.2)	0.004*
Event/party at private residence	152 (7.5)	17 (7.5)	135 (7.5)	0.289
Licensed venue	99 (4.9)	8 (3.5)	91 (5.0)	0.089
Other	152 (7.5)	13 (5.7)	139 (7.7)	0.001*
Missing = 128				
Arrival mode, *n* (%)				0.353
Ambulance	1669 (81.7)	192 (84.6)	1477 (81.3)	
Private transport	204 (10.0)	22 (9.7)	182 (10.0)	
Police/correctional services	149 (7.3)	10 (4.4)	139 (7.7)	
Other	22 (1.1)	3 (1.3)	19 (1.1)	
Missing = 0				
ATS, *n* (%)				0.012*
ATS 1	786 (38.6)	78 (34.5)	708 (39.1)	—
ATS 2	913 (44.9)	94 (41.6)	819 (45.3)	0.800
ATS 3	293 (14.4)	49 (21.7)	244 (13.5)	0.002
ATS 4–5	43 (2.1)	5 (2.2)	38 (2.1)	0.717
Missing = 9				
First GCS, *n* (%)				0.272
3–8	752 (37.3)	78 (34.4)	674 (37.6)	
9–12	328 (16.3)	45 (19.8)	283 (15.8)	
13–15	939 (46.5)	104 (45.8)	835 (46.6)	
Missing = 25				
Outcome				
ED LOS in hours, median (Q1–Q3)	4.4 (2.4–8.2)	5.4 (2.6–9.2)	4.3 (2.4–8.0)	0.926
ED disposition, *n* (%)				0.509
Short stay or observation ward	813 (40.0)	89 (39.4)	724 (40.0)	
Discharge home	685 (33.7)	69 (30.5)	616 (34.1)	
Intensive care unit	287 (14.1)	42 (18.6)	245 (13.5)	
Discharge against medical advice	79 (3.9)	8 (3.5)	71 (3.9)	
General hospital ward	75 (3.7)	9 (4.0)	66 (3.7)	
Psychiatry ward/mental health unit	52 (2.6)	6 (2.7)	46 (2.5)	
Other	44 (2.2)	3 (1.3)	41 (2.3)	
Missing = 9				

Abbreviations: ATS, Australasian Triage Scale; ED, emergency department; GCS, Glasgow Coma Scale; LOS, length of stay; NPS, new psychoactive substance.

* *p*‐value is significant (< 0.05).

### 3.2 Analytically Confirmed Drug Exposure

**FIGURE 2:**
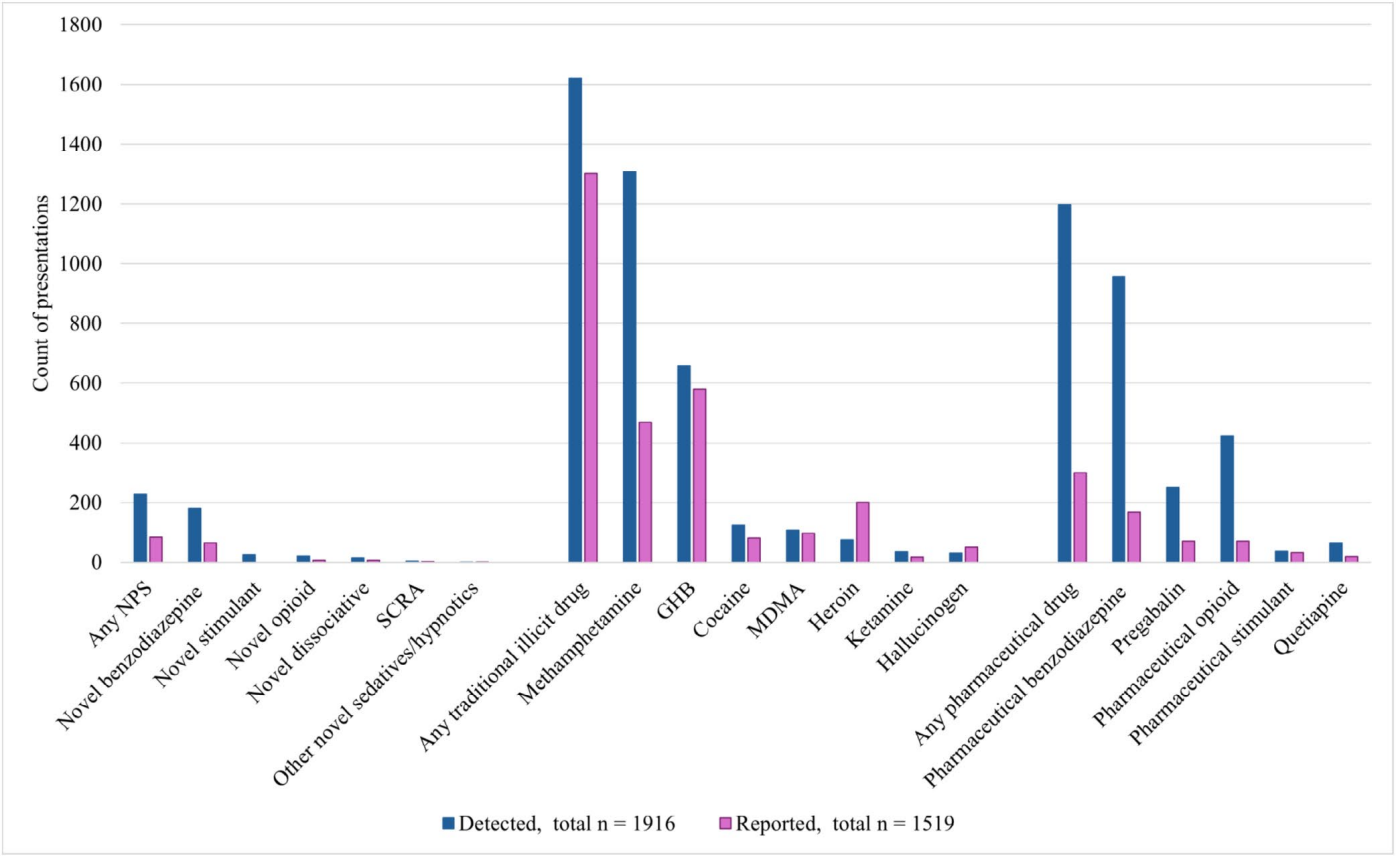
Patient‐reported versus analytically detected drug exposure in 2044 emergency department presentations included in the Emerging Drugs Network of Australia clinical registry. GHB, gamma‐hydroxybutyrate; MDMA, 3,4‐methylenedioxymethamphetamine; NPS, new psychoactive substance; SCRA, synthetic cannabinoid receptor agonist.

Analytical testing confirmed the presence of at least one pharmaceutical, traditional illicit drug, and/or NPS in 1916 (93.7%) presentations (Figure 2 and Table S3). The number of different substances detected in a single presentation ranged from one to 10, with the majority having two or more (73.9%). NPS were detected in 11.8% (227/1916) of presentations with detected drug(s), with up to six different NPS in a single presentation. Novel benzodiazepines were the most frequently detected NPS (*n =* 181, 79.7%), followed by novel stimulants (*n =* 24, 10.6%) and novel opioids (*n =* 21, 9.3%) (Figure 2). Traditional illicit drugs were detected in 1621 (84.6%) presentations. Methamphetamine was the most frequently detected traditional illicit drug, followed by GHB, cocaine and MDMA (Figure 2 and Table S3). Pharmaceutical drugs were detected in 1197 (62.5%) presentations. The most frequent pharmaceutical drug detections were benzodiazepines, opioids and pregabalin.

### 3.3 Patient‐Reported Drug Exposure

Any drug exposure was reported in 88.1% (1800/2044) of presentations. Of these, 74.3% (1519/2044) specified at least one drug within the categories reported in this study (Figure 2 and Table S3). Drug exposure was actively denied in 11.9% (244/2044) of presentations. NPS were reported in 5.6% (85/1519) of all presentations with a specified drug exposure, with novel benzodiazepines being the most frequently reported (*n =* 66, or 77.6% of all presentations with patient‐reported NPS exposure) (Figure 2 and Table S3). For traditional illicit drugs, GHB, methamphetamine and heroin were most frequently reported. The most frequently reported pharmaceuticals were benzodiazepines, pregabalin and opioids (Figure 2 and Table S3). The number of different patient‐reported substances in a single presentation ranged from one to six, with most patients reporting only one substance (55.5%, 1135/2044).

### 3.4 Accuracy of Patient‐Reported Exposure

Complete agreement between patient‐reported and analytically confirmed drug exposures was observed in 14.3% of ED presentations (293/2044). For the subgroup of patients with at least one confirmed NPS, this was 2.6% (6/227). For individual drug types, agreement between analytically confirmed and patient‐reported NPS exposure was poor (*κ* = 0.38). The strongest agreement was found for novel benzodiazepines (*κ* = 0.42) and the poorest for novel dissociatives (*κ* = 0.09). Although the specificity (98.9%, CI 98.3, 99.3%) and LR+ for patient‐reporting of all NPS sub‐types was excellent, the sensitivity of patient‐reporting was very poor, ranging from 7.1% (CI 0.1, 16.2%) for novel dissociatives to 30.9% (CI 24.3, 38.2%) for novel benzodiazepines (Table 2).

**TABLE 2:** Accuracy of patient‐reported exposure to NPS, traditional illicit and pharmaceutical drugs.

Drug	Sensitivity, % (95% CI)	Specificity, % (95% CI)	LR+, (95% CI)	LR−, (95% CI)	*κ*, (95% CI)
NPS^a^					
Any NPS	28.6 (22.9, 35.0)	98.9 (98.3, 99.3)	26.0 (16.1–42.1)	0.7 (0.6–0.8)	0.38 (0.31–0.45)
Novel benzodiazepine	30.9 (24.3, 38.2)	99.4 (99.0–99.7)	57.6 (29.9–111.0)	0.7 (0.6–0.0.8)	0.42 (0.35–0.50)
Novel opioid	19.1 (5.5–41.9)	99.9 (99.6–100)	128.4 (30.6–538.8)	0.8 (0.7–1.0)	0.28 (0.06–0.50)
Novel dissociative	7.1 (0.2–33.9)	99.7 (99.4–99.9)	24.2 (3.1–187.9)	0.9 (0.8–1.1)	0.09 (−0.08–0.26)
Traditional illicit drugs					
Any traditional illicit drug	75.7 (73.5–77.8)	78.0 (73.9–81.5)	3.4 (2.9–4.1)	0.3 (0.3–0.4)	0.44 (0.39–0.48)
GHB	76.6 (73.1–79.8)	94.5 (93.1–95.5)	13.8 (11.1–17.2)	0.2 (0.2–0.3)	0.73 (0.70–0.77)
Methamphetamine	34.3 (31.7–36.9)	97.0 (95.5–98.0)	11.5 (7.6–17.5)	0.7 (0.6–0.7)	0.25 (0.22–0.28)
Heroin	76.0 (64.8–85.1)	92.7 (91.5–93.8)	10.4 (8.5–12.7)	0.3 (0.2–0.4)	0.38 (0.31–0.45)
MDMA	60.2 (50.3–69.5)	98.4 (97.7–98.8)	36.4 (25.0–53.1)	0.4 (0.3–0.5)	0.62 (0.54–0.70)
Cocaine	45.2 (36.2–54.3)	98.7 (98.1–99.1)	34.7 (22.4–53.6)	0.6 (0.5–0.7)	0.52 (0.44–0.61)
Hallucinogen	75.0 (56.6–88.5)	98.7 (98.1–99.1)	55.9 (36.6–85.5)	0.3 (0.1–0.5)	0.57 (0.44–0.70)
Ketamine	22.2 (10.1–39.2)	99.5 (99.1–99.7)	44.6 (18.7–106.4)	0.8 (0.7–0.9)	0.29 (0.13–0.45)
Pharmaceutical drugs					
Any pharmaceutical drug	21.8 (19.5–24.3)	97.2 (95.8–98.1)	7.7 (5.1–11.6)	0.8 (0.8–0.8)	0.16 (0.14–0.19)
Pharmaceutical benzodiazepine	15.3 (13.0–17.7)	97.9 (96.8–98.6)	7.2 (4.7–11.1)	0.9 (0.8–0.9)	0.14 (0.11, 0.16)
Pregabalin	19.5 (14.8–25.0)	98.8 (98.2–99.2)	15.9 (9.8–25.9)	0.8 (0.8–0.9)	0.27 (0.20, 0.33)
Pharmaceutical opioid	10.2 (7.6–13.5)	98.3 (97.5–98.8)	5.9 (3.7–9.4)	0.9 (0.9–1.0)	0.12 (0.08, 0.16)
Pharmaceutical stimulant	15.8 (0.1–0.3)	99.6 (99.2–99.8)	39.6 (14.4–108.6)	0.8 (0.7–1.0)	0.22 (0.07, 0.38)
Quetiapine	21.5 (12.3–33.5)	99.8 (99.4–99.9)	85.2 (31.7–229.6)	0.8 (0.7–0.9)	0.32 (0.19, 0.45)

Abbreviations: CI, confidence interval; GHB, gamma‐hydroxybutyrate; LR−, negative likelihood ratio, (1‐sensitivity)/specificity; LR+, positive likelihood ratio, sensitivity/(1‐specificity); MDMA, 3,4‐methylenedioxymethamphetamine; NPS, new psychoactive substance; *κ*, Cohen's kappa.

^a^ Values for novel stimulants, synthetic cannabinoid receptor agonists, other novel sedatives/hypnotics, and unassigned novel psychoactive substances were not calculated due to insufficient counts.

Agreement between analytically confirmed and patient‐reported exposure was fair to good for several traditional illicit drugs, with kappa values of 0.73 for GHB, 0.62 for MDMA, and 0.57 for hallucinogens (Table 2). For other illicit drugs, agreement was poor, ranging from 0.25 (methamphetamine) to 0.52 (cocaine). For pharmaceutical drugs, agreement was universally poor, ranging from 0.12 to 0.32 (Table 2). Whereas specificity and LR+ of patient‐reporting were generally high for all traditional illicit drugs and pharmaceutical drugs, sensitivity varied and was lowest for pharmaceutical drugs.

### 3.5 Factors Associated With Discrepancy Between Reported and Analytically Confirmed Exposure

Discrepancy between reported and analytically confirmed exposure was present in 78.4% (1603/2044) of ED presentations; 74.5% (1522/2044) had more drugs detected than reported (Table 3). Univariate regression analyses identified older age as a factor associated with a higher odds of discrepancy (OR 1.04, CI 1.02, 1.05, *p* < 0.001). Patients attending the ED from a licensed venue (i.e., premises which are authorised to serve alcohol) or arriving by private transport or police/correctional services had significantly lower odds of discrepancy (*p* < 0.05). The odds of discrepancy were also lower in presentations with a GCS between 13 and 15 compared to presentations with a GCS between 3 and 8, and in presentations with higher acuity triage scores (*p* < 0.05) (Table 3). Of these factors, age remained independently associated with a higher odds of discrepancy. Attendance from a licensed venue, arrival by private transport, and higher initial GCS remained independently associated with a lower odds of discrepancy (Table 3).

**TABLE 3:** Univariate and multivariate logistic regression analysis for at least one false negative report.

	Discrepancy between reported and confirmed exposure (yes vs. no)		Univariate logistic regression			Multivariate logistic regression		
	Yes, *n* = 1603	No, *n* = 441	OR	95% CI	*p*	OR	95% CI	*p*
Demographic features								
Male sex, *n* (%)	1028 (64.1)	292 (66.2)	—	—	—	—	—	—
Female sex, *n* (%)	574 (35.8)	149 (33.8)	1.09	0.88, 1.37	0.729			
Missing = 1								
Age in years, median (Q1–Q3)	34 (27–42)	29 (23–39)	1.04	1.02, 1.05	< 0.001*	1.03	1.02, 1.04	< 0.001*
Missing = 8								
ED presentation characteristics								
Setting, *n* (%)					< 0.001*			
Primary residence	600 (37.4)	168 (38.1)	—	—	—	—	—	—
Public environment	616 (38.4)	129 (29.3)	1.34	1.04, 1.73	0.025*	1.28	0.98, 1.67	0.070
Event/party private residence	112 (7.0)	40 (9.1)	0.78	0.53, 1.18	0.233	0.84	0.56, 1.30	0.411
Licensed venue	58 (3.6)	41 (9.3)	0.40	0.26, 0.62	< 0.001*	0.44	0.28, 0.70	< 0.001*
Other	114 (7.1)	38 (8.6)	0.84	0.57, 1.27	0.399	0.91	0.60, 1.40	0.656
Missing = 128								
Arrival mode, *n* (%)					< 0.001*			
Ambulance	1344 (83.8)	325 (73.7)	—	—	—	—	—	—
Private transport	137 (8.5)	67 (15.2)	0.49	0.36, 0.68	< 0.001*	0.68	0.49, 0.97	0.029*
Police/correctional services	106 (6.6)	43 (9.8)	0.60	0.41, 0.87	0.007*	0.74	0.50, 1.12	0.157
Other	16 (10.0)	6 (13.6)	0.65	0.26, 1.81	0.363	0.57	0.22, 1.68	0.222
Missing = 0								
ATS, *n* (%)**					< 0.001*			
ATS 1	659 (41.1)	127 (28.8)	—	—	—			
ATS 2	696 (43.4)	217 (49.2)	0.62	0.48, 0.79	< 0.001*			
ATS 3	212 (13.2)	81 (18.4)	0.50	0.37, 0.70	< 0.001*			
ATS 4–5	28 (1.8)	15 (3.4)	0.36	0.19, 0.71	0.002*			
Missing = 9								
First GCS, *n* (%)					< 0.001*			
3–8	642 (37.9)	110 (24.9)	—	—	—	—	—	—
9–12	276 (17.2)	52 (11.8)	0.91	0.64, 1.31	0.604	0.82	0.57, 1.19	0.287
13–15	666 (41.2)	273 (61.9)	0.42	0.33, 0.53	< 0.001*	0.44	0.33, 0.57	< 0.001*
Missing = 25								

Abbreviations: ATS, Australasian Triage Scale; CI, confidence interval; ED, emergency department; GCS, Glasgow Coma Scale; OR, odds ratio.

* *p*‐value is significant.

** Although ATS was univariately significant, it was excluded from the final multivariate model due to high correlation with initial GCS.

## 4 Discussion

Our study reveals that in a large‐scale cohort of patients presenting to the ED with severe and/or unusual intoxications, a significant discrepancy exists between reported and analytically confirmed drug exposure for both traditional illicit drugs and NPS.

Poor agreement between patient‐reported drug exposure and analytical confirmation has been reported previously for several illicit and pharmaceutical drugs. Consistent with our findings, ElMehy et al. [14] reported fair agreement for cocaine (*k* = 0.51) but poor agreement for the pharmaceuticals pregabalin (*k* = 0.46) and methadone (*k* = 0.25). However, we observed lower agreement for methamphetamine and heroin compared to previous studies [14, 25]. Low frequency of patient‐reported methamphetamine exposure may reflect the high prevalence of use in the Australian population [26], and stigma experienced by people who use methamphetamine [27, 28]. Its long elimination half‐life may also explain the discrepancy between patient‐reported and confirmed methamphetamine exposure in the current study, as patients may not perceive methamphetamine as a contributing factor to their ED presentation despite remaining detectable in biological samples [29]. Higher agreement for heroin reported previously [25] may relate to variation in testing methodologies and (metabolite) detection criteria, including morphine/opiates besides 6‐monoacetylmorphine. Our results are based on a conservative definition for identifying heroin‐positive presentations (i.e., 6‐monoacetylmorphine), which has a short elimination half‐life. We did not utilise a broader criterion of co‐detected codeine and morphine as concentration data was inconsistently reported, therefore ratio calculations could not be established. While GHB was frequently detected in our cohort, this is not representative of the broader Australian population, where reported past‐year use remains low at approximately 0.2% [26]. Our results likely reflect EDNA's purposive sampling criteria, but also support evidence of increasing GHB‐related harms across multiple indicators in Australia (e.g., hospitalisations, deaths and treatment episodes) [30, 31].

The Euro‐DEN plus network reported that NPS were identified in 7.6% of recreational drug toxicity ED presentations across Europe, compared to our 11.8%. However, only one out of every five Euro‐DEN plus presentations was analytically tested and inclusions mostly relied on self‐report [32]. A positive patient report of NPS exposure in EDNA increased the likelihood of its detection. However, negative self‐reporting cannot be used to rule out exposure.

Our findings of frequent underreporting of NPS are consistent with the often unintentional nature of exposure to these substances [33]. Unintentional exposure to potent NPS through substitution and/or adulteration of traditional illicit and pharmaceutical drugs is a growing public health concern [4, 18]. The substantial rise in counterfeit benzodiazepine detections and related overdoses in Australia, including cases where multiple novel benzodiazepines were detected in single tablets sold as Xanax, Mylan or generic alprazolam [8, 34] have highlighted increased harm from counterfeit medications circulating in the community [35]. The emergence of potent synthetic opioids, such as nitazenes, also poses a particular risk in circumstances of unknown exposure [9, 21]. Multiple government‐issued public drug alerts directly informed by EDNA were disseminated during the current study period [36, 37]. These alerts provide timely and objective information on drugs of concern circulating in the community, including analytically confirmed compounds, clinical effects and harm reduction advice.

Discordance between the high proportion of presentations with multiple confirmed drug exposures, despite patients most often reporting single exposures, provides important insight into underreported polydrug exposure. In addition to unintentional exposure, patients may only report the drug(s) perceived as most clinically relevant to their current ED presentation or omit information on drugs taken days beforehand that may still be identified through blood analysis. This may also reflect the complexities of drug pharmacokinetics and the timing of sample collection. Drugs with prolonged elimination half‐lives may remain detectable in blood after their psychoactive effects have subsided, leading to detection despite the patient not considering the drug relevant to their acute presentation. To obtain more accurate information, particularly regarding polydrug exposures, clinicians need to adopt taking a more comprehensive drug exposure history. This includes asking specific questions on both recent and longer‐term exposure.

Our finding that NPS were more frequently detected in younger male patients aligns with trends observed in European EDs [32]. The current study also revealed an interesting relationship between age, NPS confirmation and discrepancy between reported and analytically confirmed NPS exposure; NPS were more frequently detected in younger patients, but older age was associated with a larger discrepancy between reported and confirmed drug exposure. The ED context and data parameters of the current study limit our ability to draw meaningful conclusions regarding these findings. Available evidence demonstrates younger individuals are more likely to purchase drugs online, including from social media apps or websites. Further exploration into demographic characteristics and engagement with online vendors is needed to better inform preventive and harm reduction strategies in an increasingly accessible online drug market [6].

Significant barriers to accurate self‐reporting of illicit drug exposure also include the stigma experienced by people who use drugs and the criminalisation of drug use [27, 28]. Fear of legal and social consequences and mistrust of healthcare providers may prevent patients from disclosing drug use to clinical staff [38]. Disclosure of drug exposure by patients can be improved by clinicians demonstrating non‐judgemental attitudes and explicitly reassuring patients about confidentiality. From a broader policy perspective, opportunities for community members to access drug checking services and receive tailored harm reduction advice have demonstrated both immediate impacts on drug use intentions and behaviour [39, 40], and benefits that extend beyond the service interaction [41]. Greater availability and access to fixed‐site and event‐based drug checking services may potentially prevent some ED presentations in the first place. Future research should explore awareness, intentions, and experiences of NPS exposure through qualitative enquiry with people who use drugs and evaluate the role of drug checking services in preventing and reducing drug‐related harm in the community.

From a clinical perspective, this study highlights that NPS and polydrug intoxication should be considered, even in patients who do not report any or multiple drug exposures. Objective evidence of NPS and illicit drugs involved in ED presentations, coupled with the associated clinical profiles, should be utilised to improve clinician capacity to effectively assess and manage patients presenting with acute toxicity. In certain circumstances, access to rapid toxicology results may also provide additional intervention guidance in cases requiring prolonged management of unknown toxicity [42].

### 4.1 Limitations

The current study has several limitations. First, patient‐reported drug exposure information was drawn from ambulance and medical records and therefore dependent on the quality and completeness of clinical documentation and accurate interpretation when entering data into the EDNA Clinical Registry. Given other sources of reported drug exposure(s) are also documented in medical records, our findings rely on the ability to distinguish patient‐reported data from other sources during medical record review and data entry, which may be unclear. The exclusion of cannabis due to interstate variability in laboratory testing protocols likely results in an underrepresentation of both reported and detected exposures. Reduced consciousness associated with acute intoxication combined with the potential for unintentional drug exposure further impacts the reliability of patient‐reported data. Additionally, no interactions were tested between drug categories, and therefore interpretation may be limited. Our findings are based on a sentinel group of participating EDs and a small subset of total drug‐related ED presentations, limiting generalisability to the broader network of Australian EDs and to other settings and population groups. Finally, differences in data collection procedures across states may have introduced variability that could not be controlled for in this analysis.

## 5 Conclusion

Poor agreement between analytically confirmed and patient‐reported drug exposure highlights the need for continued partnerships between EDs, clinical toxicologists and forensic laboratories to enable identification of the specific substances involved in acute intoxication. Especially during NPS‐related cluster events, where timely detection plays an important role, rapid access to toxicology results can inform prompt public health drug alerts and other targeted harm reduction responses.

## Author Contributions

Each author certifies that their contribution to this work meets the standards of the International Committee of Medical Journal Editors.

## Funding

EDNA is supported by a 5‐year National Health and Medical Research Council (NHMRC GNT2001107, ROR https://ror.org/011kf5r70). Additional state‐specific funding from the Government of Western Australia (East Metropolitan Health Service, the Mental Health Commission and the Department of Health) and the Government of Victoria, Department of Health, supported the current work. MJN was supported by a Marco Polo Grant from the University of Groningen.

## Conflicts of Interest

The authors declare no conflicts of interest.

## Supporting information


**Table S1:** Detected NPS, traditional illicit drugs and pharmaceutical drugs.

**Table S2:** Categorisation of patient‐reported drug exposure recorded as free‐text in ambulance and medical records.

**Table S3:** Patient‐reported versus analytically confirmed drug exposure in 2044 ED presentations included in the EDNA Clinical Registry.

## Data Availability

The data that support the findings of this study are available from the corresponding author upon reasonable request.
