# Supplementary material for: Accuracy of Patient‐Reported Exposure to New Psychoactive Substances and Other Illicit Drugs in Australian Emergency Departments: Findings From the Emerging Drugs Network of Australia
Source: Drug Alcohol Rev. 2026 Jun 15;45(5):e70193. doi: 10.1111/dar.70193 (PMC13269661; doi:10.1111/dar.70193)
Supplement: Supplementary file 1 — Table S1: Detected NPS, traditional illicit drugs and pharmaceutical drugs. [file DAR-45-0-s003.docx]

**Supplementary Table 1. Detected NPS, traditional illicit drugs and pharmaceutical drugs.**

| **Type** | **Drug/*metabolite* detected** | **n** |
| --- | --- | --- |
| **NPS** |  |  |
| Sedatives/Hypnotics:  Novel benzodiazepines | Bromazolam | 130 |
|  | Clonazolam | 46 |
|  | *8-Aminoclonazolam* | *35* |
|  | Desalkylgidazepam | 21 |
|  | Flubromazepam | 20 |
|  | Nitrazolam | 16 |
|  | Phenazolam | 11 |
|  | Etizolam | 9 |
|  | Phenazepam | 9 |
|  | Flualprazolam | 8 |
|  | Deschloroetizolam | 4 |
|  | Nimetazepam | 1 |
| Other Sedatives/hypnotics | Dihydromethysticin | 2 |
|  | Dihyodrokavain | 2 |
|  | Kavain | 1 |
| Novel stimulants | N,N-dimethylpentylone | 15 |
|  | *Pentylone* | *14* |
|  | 2,5-dimethox-4-bromophenethylamine | 1 |
|  | 2-methyl-4'-(methylthio)-2-morpholinopropiophenone | 1 |
|  | 3,4-methylenedioxydimethylamphetamine | 1 |
|  | 3,4-methylenedioxyethylamphetamine | 1 |
|  | 4-fluoroamphetamine | 1 |
|  | 4-fluoromethamphetamine | 1 |
|  | Cathine | 1 |
|  | Dibutylone | 1 |
|  | Dimethylone | 1 |
|  | Eutylone | 1 |
|  | Ethylphenidate | 1 |
|  | Methylone | 1 |
| Synthetic opioids | Protonitazene | 13 |
|  | Metonitazene | 2 |
|  | N-pyrrolidino etonitazene | 2 |
|  | 2-naphthyl U-47700 | 1 |
|  | Desocodeine | 1 |
|  | Isotonitazene | 1 |
|  | 7-hydroxymitragynine | 1 |
|  | N-pyrrolidino protonitazene | 1 |
| Dissociatives | 2-fluoro-2-oxo-PCE | 12 |
|  | 3-hydroxyphencyclidine | 2 |
|  | Deschloroketamine | 1 |
| SCRAs | 5F-CUMYL-PINACA | 3 |
|  | XLR-11 | 1 |
| Unassigned* | Mitragynine (Kratom) | 2 |
|  | Mescaline | 1 |
| **Traditional illicit drugs** |  |  |
| Methamphetamine | Methylamphetamine | 1307 |
| GHB | Gamma-hydroxybutyrate | 657 |
|  | 1,4-butanediol | 4 |
| Cocaine | Cocaine | 124 |
|  | *Benzoylecgonine* | *111* |
|  | *Ecgonine methyl ester* | *47* |
|  | *Cocaethylene* | *13* |
| MDMA | 3,4-methylenedioxymethamphetamine | 100 |
|  | *3,4-methylenedioxyamphetamine^a^* | 72 |
| Heroin | 6-monoacetylmorphine | 75 |
|  | Acetylcodeine | 1 |
| Amphetamine | Amphetamine^a^ | 69 |
|  | Dimethylamphetamine | 3 |
| Ketamine**^b^** | Ketamine | 36 |
|  | *Norketamine* | *32* |
| Hallucinogens | Lysergic acid diethylamide | 25 |
|  | Psilocine | 5 |
|  | Dimethyltryptamine | 2 |
| **Pharmaceutical drugs** |  |  |
| Pharmaceutical benzodiazepines | Diazepam | 670 |
|  | *Nordiazepam^a^* | *72* |
|  | Clonazepam | 172 |
|  | *7-aminoclonazepam* | *148* |
|  | Midazolam | 119 |
|  | *Alpha-hydroxymidazolam* | *35* |
|  | Lorazepam | 50 |
|  | Alprazolam | 47 |
|  | Oxazepam^a^ | 23 |
|  | Nitrazepam | 16 |
|  | *7-aminonitrazepam* | *14* |
|  | Temazepam^a^ | 5 |
|  | *N-desalkylflurazepam* | *2* |
|  | Flunitrazepam | 1 |
|  | *7-aminoflunitrazepam* | *1* |
| Pharmaceutical opioids | Codeine | 218 |
|  | *Norcodeine* | *5* |
|  | Methadone | 66 |
|  | Tramadol | 54 |
|  | *O-desmethyltramadol* | *44* |
|  | *N-desmethyltramadol* | *1* |
|  | Fentanyl | 4 |
|  | Buprenorphine | 33 |
|  | *Norbuprenorphine* | *7* |
|  | Oxycodone | 25 |
|  | *Noroxycodone* | *1* |
|  | Tapentadol | 23 |
|  | Dihydrocodeine^a^ | 22 |
|  | Hydrocodone | 9 |
|  | Methorphan | 8 |
|  | Dihydromorphine^a^ | 2 |
|  | Oxymorphone | 2 |
| Pregabalin | Pregabalin | 251 |
| Quetiapine | Quetiapine | 65 |
|  | *Norquetiapine* | *4* |
| Pharmaceutical stimulant | Methylphenidate | 8 |
|  | Phentermine | 6 |
|  | Ritalinic acid^a^ | 1 |

NPS = new psychoactive substance, SCRA = synthetic cannabinoid receptor agonist, GHB = gamma-hydroxybutyrate, MDMA = 3,4-methylenedioxymethamphetamine.

^a^May represent either a parent drug or a metabolite.

^b^Ketamine was classified as a traditional illicit drug if there was no documented evidence of medical administration pre-hospital and/or in the ED.
