# Supplementary material for: Accuracy of Patient‐Reported Exposure to New Psychoactive Substances and Other Illicit Drugs in Australian Emergency Departments: Findings From the Emerging Drugs Network of Australia
Source: Drug Alcohol Rev. 2026 Jun 15;45(5):e70193. doi: 10.1111/dar.70193 (PMC13269661; doi:10.1111/dar.70193)
Supplement: Supplementary file 2 — Table S2: Categorisation of patient‐reported drug exposure recorded as free‐text in ambulance and medical records. [file DAR-45-0-s001.docx]

**Supplementary Table 2. Categorisation of patient-reported drug exposure recorded as free-text in ambulance and medical records.**

| **Type** | **Patient-reported drug exposure*** |
| --- | --- |
| **NPS** |  |
| Sedatives/Hypnotics:  Novel benzodiazepines | Benzos |
|  | Street Xanax |
|  | Bromazolam |
|  | Etizolam |
|  | Mylan |
|  | Alprazolam but not prescription |
|  | Street Alrazolam |
|  | Unknown pressed xanax |
|  | Xanax and Valium |
|  | Pink Xanax |
| Other Sedatives/hypnotics | Kava |
| Synthetic opioids | Protonitazene |
|  | Etonitazene |
|  | Proton |
|  | Street opioids |
| Dissociatives | 2C-B |
|  | NBOM |
|  | PCP |
| SCRAs | Kronic |
|  | Synthetic weed |
| Unassigned | Mescaline |
| **Traditional illicit drugs** |  |
| Hallucinogens | LSD |
|  | Acid/Acid Tabs |
|  | Magic Mushrooms |
|  | Mushrooms |
|  | Psilocybin |
|  | Psilocybin mushroom powder |
|  | Shrooms |
|  | DMT |
|  | Mushroom chocolate |
| **Pharmaceutical drugs** |  |
| Pharmaceutical benzodiazepines | Alprazolam |
|  | Clonazepam |
|  | Diazepam |
|  | Lorazepam |
|  | Nitrazepam |
|  | Oxazepam |
|  | Serepax |
|  | Temazepam |
|  | Valium |
| Pharmaceutical opioids | Codeine |
|  | Buprenorphine/Suboxone |
|  | Fentanyl |
|  | Kapamol |
|  | Methadone |
|  | Morphine |
|  | Oxycodone |
|  | Oxycontin |
|  | Oxynorm |
|  | Panadeine forte |
|  | Tapentadol |
|  | Tramadol |
| Pharmaceutical stimulant | Adderall |
|  | Atomexitine |
|  | Dextromethorphan |
|  | Methylphenidate |
|  | Modafinil |
|  | Ritalin |

NPS = new psychoactive substance, SCRA = synthetic cannabinoid receptor agonist.

*This data is based on free-text information entered into the EDNA Clinical Registry as patient-reported drug exposure; it does not represent direct patient-reports.
