# Supplementary material for: Accuracy of Patient‐Reported Exposure to New Psychoactive Substances and Other Illicit Drugs in Australian Emergency Departments: Findings From the Emerging Drugs Network of Australia
Source: Drug Alcohol Rev. 2026 Jun 15;45(5):e70193. doi: 10.1111/dar.70193 (PMC13269661; doi:10.1111/dar.70193)
Supplement: Supplementary file 3 — Table S3: Patient‐reported versus analytically confirmed drug exposure in 2044 ED presentations included in the EDNA Clinical Registry. [file DAR-45-0-s002.docx]

**Supplementary Table 3.** **Patient-reported versus analytically confirmed drug exposure in 2044 ED presentations included in the EDNA Clinical Registry.**

| **Drug** | **Detected**  **n = 1916 (93.7%)** | **Reported**  **n = 1519 (74.3%)** |
| --- | --- | --- |
| **NPS** | 227 (11.8) | 85 (5.6) |
| Novel benzodiazepine | 181 (79.7) | 66 (77.6) |
| Novel stimulant | 24 (10.6) | 0 |
| Novel opioid | 21 (9.3) | 7 (8.2) |
| Novel dissociative | 14 (6.2) | 7 (8.2) |
| SCRA | 4 (1.8) | 3 (3.5) |
| Other novel sedatives/hypnotics | 2 (0.9) | 1 (1.2) |
| Unassigned | 3 (1.3) | 1 (1.2) |
| **Traditional illicit drugs** | 1621 (84.6) | 1302 (85.7) |
| Methamphetamine | 1307 (80.6) | 470 (36.1) |
| GHB | 657 (40.5) | 580 (44.5) |
| Cocaine | 124 (7.6) | 81 (6.2) |
| MDMA | 108 (6.7) | 97 (7.6) |
| Heroin | 75 (4.6) | 201 (15.4) |
| Ketamine | 36 (2.2) | 18 (1.4) |
| Hallucinogen | 32 (2.0) | 51 (3.9) |
| **Pharmaceutical drugs** | 1197 (62.5) | 300 (19.7) |
| Pharmaceutical benzodiazepine | 957 (79.9) | 169 (56.3) |
| Pregabalin | 251 (2.1) | 71 (23.7) |
| Pharmaceutical opioid | 423 (3.5) | 71 (23.7) |
| Pharmaceutical stimulant | 38 (3.2) | 34 (11.3) |
| Quetiapine | 65 (5.4) | 19 (6.3) |

NPS = new psychoactive substance, SCRA = synthetic cannabinoid receptor agonist, GHB = gamma-hydroxybutyrate, MDMA = 3,4-methylenedioxymethamphetamine.

Note that the total percentage of drugs reported and detected exceeds 100 due to polydrug exposure.
